# Dark Side of Performance-Oriented Competition: Dual Strain Pathways to Affective Organizational Commitment Among Female Managerial Employees

**DOI:** 10.3390/bs16091657

**Published:** 2026-09-16

**Authors:** Abdulkhamid Komil ugli Fayzullaev, Alisher Tohirovich Dedahanov, Soo Young Shin, Farkhodbek Vokhobzhon ugli Yuldashev

**Affiliations:** 1 School of Business, Yeungnam University, Gyeongsan 38541, Republic of Korea; fayzziyo@yu.ac.kr (A.K.u.F.); splendid7727@gmail.com (F.V.u.Y.); 2 School of Management, New Uzbekistan University, Tashkent 100000, Uzbekistan; a.dedahanov@newuu.uz

**Keywords:** female manager, performance-oriented competition, interpersonal strain, work overload, affective organizational commitment, job autonomy

## Abstract

Competition is widely promoted as a mechanism for enhancing employee motivation and organizational performance. However, its unintended costs for female managers remain underexplored. This study examines how performance-oriented competition (POC) affects affective organizational commitment (AOC) through workplace interpersonal strain (WIS) and work overload strain (WOS), and whether job autonomy moderates these relationships among female managerial employees. Data were drawn from the seventh wave of the Korean Women Manager Panel, a large-scale national survey in South Korea. The final sample comprised 1325 females across 413 organizations. Mediation and moderation analyses were conducted using the PROCESS macro with bootstrapped confidence intervals. Results indicate that POC is positively associated with both WIS and WOS, which are in turn negatively associated with AOC. Both forms of strain significantly mediate the relationship between competition and commitment, with the WIS pathway being stronger. Job autonomy does not affect the relationship between POC and WIS but unexpectedly strengthens the positive association between POC and WOS. These findings suggest that competitive work systems may impose hidden costs by weakening female managers’ organizational commitment. This study extends research on competitive climates and challenges the assumption that job autonomy is uniformly beneficial. Organizations should exercise caution when relying on competition and autonomy as motivational tools.

## 1. Introduction

Contemporary organizations increasingly implement competitive performance systems (e.g., tournament-style rewards, forced rankings, and rigorous performance comparisons) to stimulate effort and identify top talent (Hu & Wang, 2022). Similarly, there is a global push to increase the representation and retention of females in organizations (H. Kim & Lee, 2025). These trends may be at odds. Although competitive systems are often viewed as meritocratic and performance-enhancing, they may inadvertently disadvantage females.

Affective organizational commitment (AOC) refers to employees’ emotional attachment to, identification with, and involvement in their organization (Meyer et al., 2002). It is a critical attitude associated with lower turnover (Moreira et al., 2024; Perreira et al., 2018), greater engagement (Shen et al., 2023), and improved job performance (Reiley & Jacobs, 2019). AOC is particularly important for female employees, especially managers, whose recruitment and retention are essential to organizational success (Halliday et al., 2018; Yim et al., 2022). Research shows that females report lower organizational commitment than males (K. Y. Kim et al., 2016; Peterson et al., 2019), underscoring the need to identify workplace conditions that may undermine their commitment.

Despite growing interest in competitive work environments, research has focused primarily on motivation, engagement, and performance outcomes (Jones et al., 2017; W. J. T. Lee et al., 2022). Far less is known about whether perceived performance-oriented competition (POC) erodes AOC, particularly among female managerial employees. This gap is important. Prior research suggests that some women experience competitive work environments differently and place greater value on cooperative workplace relationships (Yun et al., 2019; Furtner et al., 2021; Miller et al., 2024). Competitive environments emphasize agentic behaviors such as rivalry and self-promotion, which may conflict with socially prescribed cooperative roles for female employees (Eagly & Karau, 2002; Yun et al., 2019). These dynamics can increase interpersonal tension and strain.

Drawing on job demands–resources (JD-R; Bakker et al., 2023a) and conservation of resources (COR) theories (Hobfoll et al., 2018), we conceptualize POC as a contextual job demand that diminishes AOC through two strain-based pathways. First, female leaders tend to place greater emphasis on social and relational resources at work, including positive interpersonal relationships, social support, and cooperation (Cho et al., 2016; Davcheva & González-Romá, 2023). By fostering rivalry and social tension, POC may erode these valued resources and generate interpersonal strain (Xu et al., 2025; Yıldız et al., 2023). Second, because females often face competing work and family responsibilities (Herrero et al., 2012; Skinner & Pocock, 2008), competition may function as an additional demand that intensifies role burden and heightens perceptions of work overload. Both forms of strain may deplete the psychological resources required to sustain affective commitment (Wright & Hobfoll, 2004).

JD-R theory further posits that job resources such as autonomy can buffer the negative effects of job demands (Demerouti, 2025). Autonomy enables employees to manage demands and reduces vulnerability to resource depletion (Bakker et al., 2023a; Hobfoll et al., 2018). Prior research suggests that autonomy may be particularly beneficial for females facing heightened demands and constraints (Halliday et al., 2018). Accordingly, we examine whether job autonomy attenuates the adverse effects of POC on female managers.

We test our hypotheses using survey data from 1325 female managerial employees in South Korea, a context where female employment remains relatively low despite high educational attainment and increasing policy attention to gender equity (The Korea Times, 2025; OECD, 2025). Although the employment rate gap between males and females has steadily narrowed and the proportion of female managers has increased over the past decade, female representation declines substantially at higher management levels (Yim et al., 2022). This setting provides insight into how organizational systems may undermine the sustainability of female careers.

This study makes three contributions. First, it extends research on competitive work environments by examining female managerial employees’ affective commitment as a key attitudinal outcome. Second, it identifies dual strain pathways (i.e., interpersonal and overload-related) through which competition erodes commitment. Third, it informs debates on job resources by testing whether autonomy retains its protective role in competitive contexts. Overall, the study offers a more critical perspective on meritocratic competition systems, showing that practices designed to identify talent may also weaken the commitment of the female managers organizations seek to retain.

## 2. Theory and Hypotheses

### 2.1. Theoretical Background

This study is grounded in JD-R and COR theories to examine the relationships among POC, employee strain, job autonomy, and AOC among female managerial employees. JD-R theory explains how physical and social aspects of the work environment, particularly job demands and resources, affect employee well-being and job-related outcomes (Bakker & Demerouti, 2024). Job demands are aspects of work that require sustained effort and may produce psychological or physiological strain, such as heavy workloads or emotionally taxing interactions. In contrast, job resources help employees achieve goals, reduce demands, or foster growth and include job autonomy, social support, and positive workplace relationships (Bakker & Demerouti, 2007). The theory’s central principle, the health-impairment process, posits that excessive demands can strain individuals and lead to adverse outcomes, whereas resources can mitigate these effects (Bakker & Demerouti, 2007). The JD-R model has been widely applied to explain strain, burnout, commitment, and performance across diverse organizational settings, including competitive environments (Bakker & Demerouti, 2007; Fehr & Koob, 2025; Fousiani et al., 2026). Within this framework, POC represents a job demand, workplace interpersonal strain (WIS) and work overload strain (WOS) represent forms of employee strain, job autonomy serves as a resource, and AOC is a key outcome.

COR theory complements JD-R by proposing that individuals seek to acquire, retain, and protect valued resources such as time, energy, relationships, and well-being (Hobfoll, 2001). Stress occurs when these resources are threatened, lost, or insufficiently replenished. Because resource loss has a greater impact than resource gain, individuals work to conserve existing resources and avoid further depletion (Hobfoll et al., 2018). COR theory has also been widely used to explain how demanding workplace conditions and competitive environments shape employees’ psychological states and work outcomes (W. J. T. Lee et al., 2022; Rai et al., 2024).

Recent studies suggest that JD-R and COR theories provide complementary perspectives on employee well-being (Bakker et al., 2023b; Demerouti, 2025). JD-R explains the relationships among demands, resources, strain, and outcomes, whereas COR clarifies the resource preservation and loss processes underlying those relationships. In this study, JD-R serves as the overarching framework, while COR provides the explanatory mechanism underlying employees’ responses to demanding work conditions and available job resources.

### 2.2. POC and WIS

A competitive work environment is generally defined as one in which employees perceive structured competition among coworkers for rewards, recognition, and status (Fletcher & Nusbaum, 2010). Similarly, a competitive psychological climate reflects the extent to which organizational rewards are perceived as contingent on peer comparisons (Brown et al., 1998). Building on these perspectives, perceived POC can be understood as employees’ assessment of the extent to which rewards and recognition depend on outperforming others.

COR theory posits that individuals strive to acquire and protect valued resources such as energy, self-esteem, and social relationships (Hobfoll et al., 2018), and that stress arises when these resources are threatened or lost (Bartram et al., 2023). In organizations, supportive coworker relationships are critical resources for managing job demands (Nguyen & Tuan, 2022). Females, including those in leadership roles, tend to place greater value on collaboration and interpersonal relationships at work (Cho et al., 2016; Davcheva & González-Romá, 2023; Konrad et al., 2000). However, in performance-oriented competitive environments, coworkers become rivals for limited rewards, undermining social support and fostering tension. As employees adopt self-protective behaviors and withhold cooperation, relationships deteriorate (Murtza & Rasheed, 2023), increasing interpersonal strain.

From a role congruity perspective, competitive environments emphasize agentic behaviors (e.g., rivalry, assertiveness, and self-promotion) that may conflict with communal role expectations typically associated with females (del Carmen Triana et al., 2024). This mismatch creates interpersonal tension as females navigate competing expectations of cooperation and competition, increasing relational strain. Competitive climates therefore disrupt collaborative norms and heighten the likelihood of social friction.

Empirical evidence supports these arguments. Workplace competition is associated with reduced advice-seeking and knowledge sharing, as well as increased secrecy (Anand et al., 2020). It also fosters envy and heightened sensitivity to peers’ performance (Yıldız et al., 2023; Wang, 2022). Accordingly, female managers who perceive higher levels of POC are more likely to experience interpersonal strain.

**Hypothesis** **1.** *POC is positively related to WIS among female managerial employees.*

### 2.3. POC and WOS

When employees perceive POC, where rewards, evaluations, and recognition depend on outperforming peers (Fletcher & Nusbaum, 2010), they face greater pressure to exert effort to secure valued outcomes. From a COR perspective, such conditions signal potential resource loss (e.g., rewards and status), prompting individuals to invest additional resources to prevent that loss (Bartram et al., 2023). Time, energy, and cognitive capacity are particularly vulnerable under these demands. Employees may work longer hours, multitask, or assume additional responsibilities to maintain a competitive edge, thereby increasing workload (Wang, 2022). This sustained effort depletes resources and heightens perceptions of work overload. Because resource loss is more salient than gain (Hobfoll et al., 2018), employees tend to overinvest effort, further intensifying depletion and strain.

These effects may be especially relevant for females, who often face dual demands from work and family responsibilities (Herrero et al., 2012; Halliday et al., 2018). The combination of competitive pressure and persistent nonwork demands can amplify perceived overload and accelerate resource depletion.

Empirical evidence supports this reasoning. Competitive environments are associated with higher job demands and greater strain, including elevated work overload (Jiandong et al., 2022; K. Y. Kim et al., 2023), consistent with COR’s loss-spiral mechanism, in which initial depletion triggers further resource loss. Accordingly, higher perceived POC is expected to increase WOS among female managers.

**Hypothesis** **2.** *POC is positively related to WOS among female managerial employees.*

### 2.4. WIS and AOC

AOC reflects employees’ emotional attachment to, identification with, and involvement in their organization (Meyer & Allen, 1991). Supportive interpersonal relationships are key job resources that foster AOC (Fehr & Koob, 2025). Frequent positive interactions, particularly those that provide emotional support, strengthen attachment and create a sense of belonging (Rabbi et al., 2026; Stark et al., 2025; Su et al., 2024). Accordingly, employees who experience constructive workplace relationships tend to exhibit higher organizational commitment (Chen & Liu, 2022).

From a COR perspective, sustaining organizational commitment requires ongoing resource investment (Wright & Hobfoll, 2004). Positive relationships with colleagues and supervisors provide critical resources, including emotional support and psychological safety, that sustain affective commitment (Fehr & Koob, 2025). When these resources are threatened or lost, as in WIS, employees conserve their remaining resources by withdrawing social and emotional investment. This withdrawal weakens their connection to the organization and reduces affective commitment. These effects may be particularly relevant for female managers, whose workplace experiences often depend heavily on social inclusion and support (Cho et al., 2016; Loes & Tobin, 2018). Consistent with this reasoning, interpersonal strain and conflict reduce employees’ sense of belonging and emotional attachment (Kacmar et al., 2012; Thomas et al., 2005).

**Hypothesis** **3.** *WIS is negatively related to AOC among female managerial employees.*

### 2.5. WOS and AOC

In organizational research, workload is increasingly conceptualized as a subjective experience shaped by individual perceptions rather than objective demands (Ekmekci et al., 2021). Accordingly, perceived workload reflects the extent to which individuals believe they lack sufficient resources to meet job requirements (Peterson et al., 1995). From a COR perspective, when job demands exceed available resources, employees expend time and energy to cope (Hobfoll et al., 2018). As work overload consumes these limited resources (Halbesleben et al., 2014), employees prioritize core tasks and reduce discretionary effort to conserve what remains. Because AOC requires sustained emotional and psychological investment, employees experiencing overload are less able or willing to maintain a strong attachment to the organization (Wright & Hobfoll, 2004).

Empirical evidence supports this mechanism. Work overload limits employees’ capacity to engage in positive workplace behaviors, even when motivation remains high (Sofyan et al., 2021). These effects may be particularly relevant for females in managerial roles, who often struggle balancing work, household, and family responsibilities (Cho et al., 2016; Y. Lee, 2017). Additional nonwork demands further deplete resources, intensify role strain, and constrain investment in work-related attitudes and relationships (Marsden et al., 1993). As a result, female managers experiencing overload are more likely to withdraw emotional investment, reducing affective commitment. Prior research supports a negative relationship between work overload and AOC, including among female-dominated samples (Ekmekci et al., 2021; Ko et al., 2022). Accordingly, WOS is expected to reduce AOC.

**Hypothesis** **4.** *WOS is negatively related to AOC among female managerial employees.*

### 2.6. Mediating Roles of WIS and WOS

Drawing on COR theory, we propose that WIS and WOS serve as mechanisms through which perceived POC undermines AOC. POC functions as a job demand that depletes resources through multiple pathways. High levels of competition increase interpersonal strain by eroding collaboration, encouraging knowledge withholding, and reducing social support (Anand et al., 2020), thereby weakening the relational resources essential for belonging and emotional connection (Jerrim & Sims, 2025). In addition, competitive environments heighten the risk of resource loss (e.g., rewards and status), prompting employees to invest greater time and effort to maintain their standing (Bartram et al., 2023). This investment contributes to work overload (Wang, 2022), further draining energy and limiting employees’ capacity to sustain affective commitment (Wright & Hobfoll, 2004).

These processes may be especially relevant for females, who often rely more heavily on social resources and face greater nonwork demands. Consequently, competition is likely to deplete both relational and personal resources among female managerial employees, intensifying interpersonal and workload strain and ultimately reducing affective commitment. Accordingly, we hypothesize the following:

**Hypothesis** **5a.** *WIS mediates the link between POC and AOC among female managerial employees.*

**Hypothesis** **5b.** *WOS mediates the link between POC and AOC among female managerial employees.*

### 2.7. Moderating Role of Job Autonomy

POC can heighten employees’ experiences of strain, both in interpersonal relationships and in perceptions of work overload. However, the effects of competitive environments are unlikely to be uniform across employees. According to the JD-R framework, job resources can buffer the negative effects of job demands (Bakker et al., 2023a). Among these resources, job autonomy—the degree of freedom and independence employees have in making decisions (Morgeson & Humphrey, 2006)—is particularly important. Job autonomy enables employees to regulate their effort, prioritize tasks, and manage social interactions, thereby reducing the likelihood that competitive pressures escalate into strain (Demerouti, 2025; Wan & Duffy, 2022).

**Figure 1:**
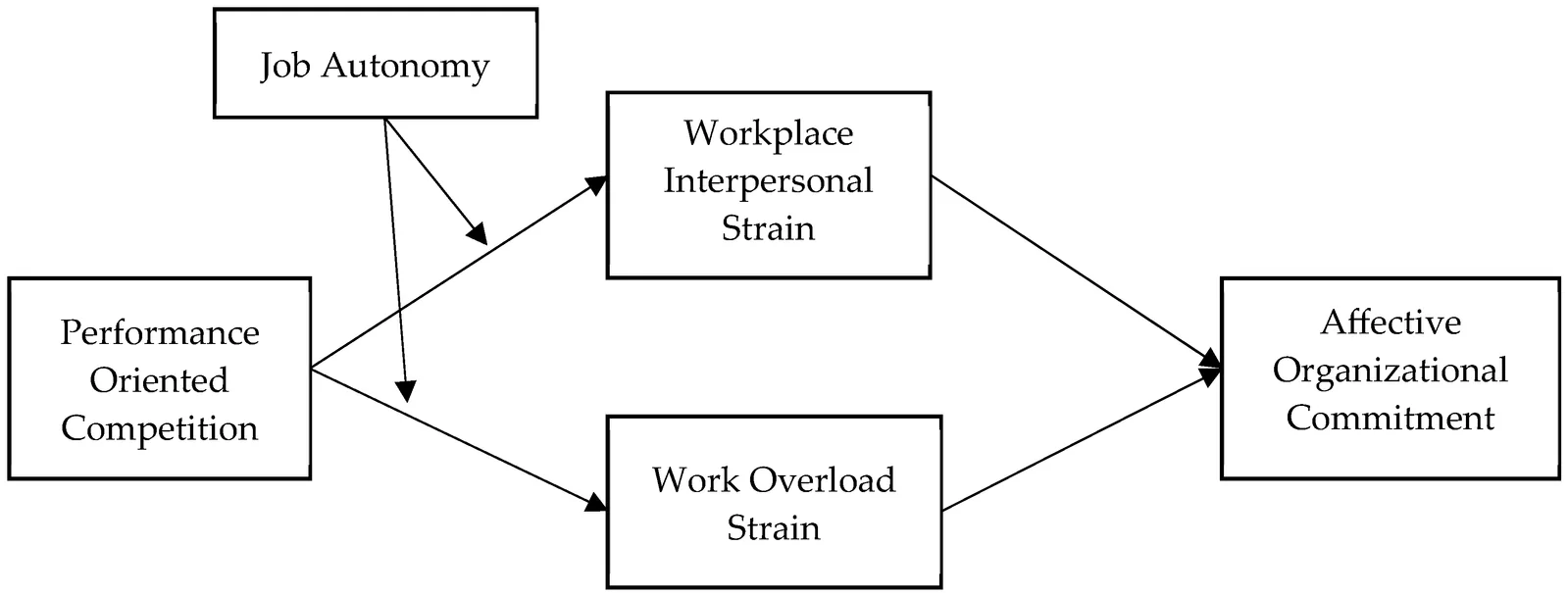
Research hypothesis model.

This buffering role may be especially relevant for female employees. Because women managers tend to rely more heavily on relational resources and face greater nonwork demands (Cho et al., 2016; Davcheva & González-Romá, 2023), autonomy provides greater control over interpersonal dynamics and helps balance competing responsibilities. As a result, autonomy may help preserve social resources and prevent the accumulation of excessive workloads under competitive conditions. Consistent with this reasoning, prior research suggests that autonomy may be particularly beneficial for women facing heightened demands (Halliday et al., 2018). Therefore, job autonomy is expected to moderate the relationship between POC and both WIS and WOS, such that these positive relationships are weaker at higher levels of autonomy. Accordingly, we propose the following hypotheses (Figure 1):

**Hypothesis** **6a.** *Job autonomy moderates the relationship between POC and WIS among female managerial employees, such that the positive relationship is weaker at higher levels of job autonomy.*

**Hypothesis** **6b.** *Job autonomy moderates the relationship between POC and WOS among female managerial employees, such that the positive relationship is weaker at higher levels of job autonomy.*

## 3. Method

### 3.1. Sample and Procedure

This study employed a quantitative research design using data from the seventh wave of the Korean Women Manager Panel, a nationally representative survey conducted biennially since 2007. A quantitative approach was selected to examine a theoretically specified model involving direct, indirect, and moderating relationships, requiring statistical assessment of the strength and significance of these associations. This panel dataset is particularly well suited to the study because it captures challenges faced by female managerial employees, including interpersonal pressure in competitive settings, demanding performance evaluations, and reliance on workplace relationships for career advancement. The large-scale panel survey provided access to a diverse sample of female employees and enabled rigorous examination of the proposed relationships.

Data were drawn from 413 companies employing at least one female manager and having 100 or more employees. The sample comprised 1325 females across functional areas, including accounting, finance, marketing, sales, production, customer service, and labor management. Respondents were nested within 413 organizations, with an average of approximately 3.21 respondents per organization. Because the study focused on individual-level relationships, the primary analyses were conducted at the individual level. Participants ranged from assistant manager to executive level (assistant manager: 21.4%; manager: 27.1%; deputy manager: 33.7%; general manager: 14.4%; executive: 3.4%). Respondents ranged in age from 27 to 65 years (M = 44.08). Regarding education, 8.3% completed high school, 14.2% graduated from college, 55.5% held a four-year university degree, 20.5% held a master’s degree, and 1.6% held a PhD. Most respondents were married (75.5%), followed by single (21.0%), divorced (2.9%), and widowed (0.6%).

### 3.2. Measurements

Unless otherwise noted, all items were measured using a five-point Likert scale (1 = “strongly disagree” to 5 = “strongly agree”). Because this study relies on a nationally administered archival survey rather than a primary dataset, several constructs were measured using concise indicators. This trade-off is common in large-scale panel surveys, where questionnaire length must be balanced against construct coverage. Prior research indicates that brief measures can demonstrate acceptable validity for concrete, clearly interpretable constructs (Bergkvist & Rossiter, 2007; Cheung & Lucas, 2014; Diamantopoulos et al., 2012; Wanous & Reichers, 1997).

POC was measured using three items assessing perceived competition based on performance outcomes. A sample item is “Performance-oriented competition is severe.” Internal consistency was acceptable (Cronbach’s α = 0.71). WIS was measured using two items capturing relational difficulty and interpersonal tension. A sample item is “I experience difficulties in my relationships with colleagues or subordinates.” Higher scores indicate greater strain (α = 0.76). WOS was measured using two items reflecting excessive workload and difficulty managing demands. A sample item is “My workload is excessive.” Higher scores indicate greater overload (α = 0.75). AOC was measured using six items adapted from prior research (e.g., Rhoades et al., 2001). A sample item is “I feel a sense of belonging and attachment to my organization.” Higher scores indicate stronger attachment (α = 0.92). Job autonomy was assessed using a single item: “I have no autonomy in my job,” reverse-coded so that higher values indicate greater autonomy. This measure captures perceived decision latitude rather than a multidimensional construct. Single-item indicators are commonly used in large-scale surveys to measure concrete perceptions (Wanous & Reichers, 1997).

Consistent with prior research (Bae & Yang, 2017; Marsden et al., 1993), age, education level, marital status, and position were included as control variables because they may influence workplace experiences, resource availability, and organizational attachment. Age was measured in years; education level was assessed on a five-point ordinal scale (1 = high school; 2 = college; 3 = bachelors; 4 = masters; 5 = PhD); marital status was dummy-coded (0 = unmarried, including single, divorced, or widowed; 1 = married); and position was assessed on a five-point ordinal scale (1 = assistant manager, 2 = manager, 3 = deputy manager, 4 = general manager, 5 = executive).

## 4. Results

We first conducted preliminary analyses using IBM SPSS 23 to assess regression assumptions, examining skewness and kurtosis to evaluate normality. Skewness ranged from −0.52 to 0.35 and kurtosis from −0.39 to 0.06, indicating that all variables met accepted normality thresholds (skewness < 3; kurtosis < 10; Kline, 2005).

We then examined descriptive statistics and correlations (Table 1). POC was positively associated with WIS (r = 0.248, *p* < 0.01) and WOS (r = 0.435, *p* < 0.01) and negatively associated with AOC (r = −0.163, *p* < 0.01). Both WIS (r = −0.309, *p* < 0.01) and WOS (r = −0.159, *p* < 0.01) were also negatively associated with AOC. Overall, these findings are consistent with the hypothesized relationships.

Before hypothesis testing, we assessed multicollinearity among the independent variables. Variance inflation factor values ranged from 1.155 to 1.360, and tolerance values ranged from 0.735 to 0.866, all of which were within acceptable ranges, indicating no multicollinearity concerns.

Although the study relies on self-reported data, which may introduce common method variance, several observations reduce concerns about construct redundancy and multicollinearity, though common method bias cannot be ruled out. The primary variables represent distinct constructs, including contextual perceptions (POC), strain experiences (WIS and WOS), job design perceptions (autonomy), and organizational attitudes (AOC). Correlations were differentiated rather than uniformly high, and multicollinearity diagnostics indicated no concerns. Nevertheless, these observations do not constitute direct evidence that common method variance has been mitigated. Future research should employ multi-source, time-lagged, or experimental designs to further address potential bias.

Given that respondents were nested within organizations, the extent of between-organization dependence was assessed by calculating intraclass correlation coefficients (ICCs; ICC (1) and ICC (2)) and design effects for each study variable. The ICC (1) values ranged from 0.036 to 0.141, the corresponding ICC (2) values ranged from 0.106 to 0.346, and design effects between 1.08 and 1.31, based on the average cluster size of 3.21 respondents per organization. All design effects were below the commonly accepted threshold of 2.0, suggesting that clustering was unlikely to have materially affected the standard errors or substantive conclusions. Accordingly, the subsequent analyses were conducted at the individual level.

We used Hayes’ PROCESS macro (SPSS v.4.2) to test direct and indirect relationships (Model 4; 5000 bootstrap samples; 95% bias-corrected confidence intervals (CIs)), controlling for age, education, marital status, and position. As shown in Table 2, POC is positively associated with WIS (*b* = 0.261, *p* < 0.001; 95% CI = 0.206, 0.315) and WOS (*b* = 0.482, *p* < 0.001; 95% CI = 0.428, 0.536), supporting Hypotheses 1 and 2. WIS (*b* = −0.249, *p* < 0.001; 95% CI = −0.298, −0.201) and WOS (*b* = −0.055, *p* < 0.05; 95% CI = −0.104, −0.005) are negatively associated with AOC, supporting Hypotheses 3 and 4.

Beyond statistical significance, the magnitude of the observed relationships varied across pathways. Although some effects were relatively modest, the effects of POC on WIS (*b* = 0.261), POC on WOS (*b* = 0.482), and WIS on AOC (*b* = −0.249) were among the strongest relationships observed. In addition, the model explained 19.1% of the variance in WOS and 18.7% of the variance in AOC.

Mediation analysis (Table 3) shows that the indirect effects of POC on AOC through WIS (*b* = −0.065; SE = 0.010, 95% CI = −0.086, −0.046) and WOS (*b* = −0.026; SE = 0.012, 95% CI = −0.051, −0.002) are both significant, indicating mediation. The direct effect of POC on AOC remains significant after the mediators are included, supporting Hypotheses 5a and 5b and indicating partial mediation. Among the control variables, age (*b* = 0.031, *p* < 0.001; 95% CI = 0.024, 0.038), marital status (*b* = 0.164, *p* < 0.001; 95% CI = 0.075, 0.253), and position (*b* = 0.061, *p* < 0.01; 95% CI = 0.024, 0.098) are positively associated with AOC.

A post hoc contrast test indicates that the interpersonal strain pathway is significantly stronger than the work overload pathway (difference −0.039; 95% CI = −0.072, −0.005), suggesting that the relational effects of competitive climates may be particularly consequential for female employees’ organizational commitment.

Moderation effects were tested using PROCESS Macro (Model 1). Continuous predictors were mean-centered using the default settings. As shown in Table 4, the interaction between POC and job autonomy does not significantly predict WIS (*b* = 0.008, SE = 0.026, 95% CI = −0.043, 0.059), indicating no moderation effect. In contrast, the interaction significantly predicts WOS (*b* = 0.056, SE = 0.025, 95% CI = 0.006, 0.105), but in a positive direction, contrary to the hypothesis.

Simple slope analysis shows that POC is positively associated with WOS at both low (−1 SD; *b* = 0.360, SE = 0.036, *p* < 0.001, 95% CI = 0.289, 0.431) and high (+1 SD; *b* = 0.462, SE = 0.035, *p* < 0.001, 95% CI = 0.393, 0.531) levels of autonomy, with the relationship being stronger at higher levels of autonomy. Collectively, these findings do not support Hypotheses 6a and 6b.

## 5. Discussion

This study examined how POC influences female managerial employees’ AOC through WIS and WOS and whether job autonomy moderates these relationships. Using a large national sample of female managers in South Korea, the findings show that practices intended to enhance performance can also generate resource-depleting experiences that undermine commitment.

Several key findings emerged. First, stronger perceived competition is associated with higher levels of both WIS and WOS, indicating that competition may be experienced as a source of relational tension and workload pressure among female managerial employees. For female managers, who often place greater value on positive workplace relationships, support, and cooperation, workplace competition may undermine these valued relational resources. Furthermore, competitive environments may increase pressure to perform, encouraging employees to exert greater effort and work longer hours. Consistent with COR theory, these findings suggest that competitive psychological climates can initiate resource-depleting processes that manifest as both interpersonal strain and work overload. This finding aligns with prior research linking competitive environments to negative interpersonal dynamics and work-role overload (To et al., 2020; Yıldız et al., 2023; Xu et al., 2025; K. Y. Kim et al., 2023).

Second, both WIS and WOS are negatively associated with AOC, suggesting that employees experiencing greater interpersonal tension or workload pressure may feel less emotionally attached to their organizations. For female managers, such strain may be particularly detrimental because it undermines valued workplace resources, including supportive relationships and the capacity to manage work demands effectively. Because females often face dual demands from work and family responsibilities, relational and workload strains may deplete the resources needed to maintain emotional attachment to their organizations. These findings are consistent with evidence linking interpersonal strain and work overload to lower AOC (Loes & Tobin, 2018; Ko et al., 2022).

Third, both WIS and WOS mediate the relationship between competition and commitment, indicating that the negative consequences of POC for AOC operate partly through strain-related processes. These findings suggest that competition may weaken female managers’ emotional attachment to their organizations by eroding the social and personal resources needed to sustain positive work experiences. Post hoc analyses further indicate that the interpersonal pathway may be stronger than the work overload pathway, suggesting that relational resource loss may be particularly consequential in competitive contexts. This finding is consistent with prior research highlighting the importance of social support and positive workplace relationships in many females’ work experiences. However, because this comparison was exploratory, the findings should be interpreted cautiously and warrant further investigation.

Finally, job autonomy did not significantly moderate the relationship between POC and WIS, suggesting that autonomy may be less effective in alleviating interpersonal tensions associated with competitive work environments. This may reflect the inherently relational nature of managerial work, where frequent coordination with supervisors, coworkers, and subordinates makes competition-induced strain difficult to resolve through individual discretion alone. Interestingly, contrary to expectations, job autonomy strengthened the relationship between POC and WOS. Although autonomy is often viewed as a job resource (Halliday et al., 2018), our results suggest that its benefits may depend on the specific demands employees face. In competitive contexts, autonomy may increase responsibility and accountability for meeting performance goals and may also heighten uncertainty (Wan & Duffy, 2022; Rudolph et al., 2025), reducing its effectiveness as a coping resource. This interpretation aligns with evidence from Korean employees indicating that autonomy may lose its benefits under high-performance pressure (Jang & Kim, 2025). Overall, these findings challenge the assumption that job autonomy is universally beneficial and suggest that its effects may vary according to the type of strain experienced in competitive work environments. The Korean organizational context should also be considered when interpreting these findings. Korean workplaces are often characterized by hierarchical structures and strong performance expectations, which may amplify competition and shape experiences of workplace strain and autonomy. Although our interpretations are grounded in JD-R and COR theories, the Korean context may influence both the intensity and manifestation of these dynamics.

### 5.1. Theoretical Contribution

This study offers several theoretical contributions. First, it extends research on competitive work climates, which has focused primarily on performance, motivation, and achievement, by demonstrating that competition can also generate attitudinal costs through reductions in female managers’ AOC. This contribution is important because commitment underpins retention, citizenship behavior, and long-term effectiveness. Systems that enhance short-term performance while weakening commitment may ultimately undermine organizational outcomes.

Second, the study adopts a demand–resource perspective to explain how competition undermines AOC. POC operates as a job demand that depletes psychological resources through WIS and WOS. Identifying these dual pathways demonstrates that competitive environments can simultaneously erode relational and energetic resources. This finding also contributes to research on females in organizations by highlighting the mechanisms through which POC may influence female managers’ AOC. Post hoc evidence further suggests that the interpersonal pathway may be stronger than the workload pathway, underscoring the potential importance of relational resource loss in competitive settings.

Finally, the unexpected moderation effect of autonomy challenges the JD-R assumption that job resources are uniformly beneficial. Under competitive pressure, autonomy may create expectations of continuous availability, self-management, and accountability, shifting from a resource to a mechanism that internalizes pressure and amplifies strain.

### 5.2. Practical Implications

The findings offer several practical implications. Organizations should exercise caution when relying on performance-based competition as a default motivational strategy. Although competition can stimulate effort, excessive emphasis on outperforming coworkers may weaken commitment among female managerial employees. A coopetitive (a term used to describe situations in which individuals, teams, or organizations compete and cooperate simultaneously) approach that integrates competition and collaboration can preserve performance benefits while reducing unintended costs (dos Santos et al., 2023). For example, replacing individual rankings with team-based goals, shared incentives, and collaborative performance metrics may mitigate negative social dynamics without sacrificing effectiveness.

Organizations should also recognize that competitive systems are not uniformly effective. Prior research suggests that demographic characteristics may influence how employees experience workplace practices (Ely & Meyerson, 2000), underscoring the importance of person–environment fit in role design and assignment (Spurk et al., 2019). Individual differences in competitiveness further shape responses to such environments (Fletcher et al., 2008; Schrock et al., 2016). Because interpersonal and workload strain link competition to reduced commitment, organizations should strengthen social support through team-building, mentoring, and cross-functional collaboration (Chiaburu & Harrison, 2008).

Organizations should not assume that autonomy alone offsets competitive pressure. Autonomy must be supported by realistic workload expectations, managerial guidance, adequate staffing, and clear priorities. Without such support, autonomy may shift the burden of managing demands onto employees, effectively creating self-managed overload (Jang & Kim, 2025).

Finally, organizations often place greater emphasis on recruitment than retention. Competitive climates that intensify strain may gradually erode female managers’ organizational attachment. These findings highlight the importance of prioritizing retention systems alongside recruitment efforts.

### 5.3. Limitations and Future Research Directions

Despite its contributions, this study has several limitations. First, the use of secondary panel data limited measurement to brief indicators rather than comprehensive multi-item scales. However, the nationally representative sample enhances external validity, and prior research supports the adequacy of brief measures in large-scale panel contexts (H. Lee & Lee, 2021; Minibas-Poussard et al., 2025). Future research should employ more comprehensive, validated scales to improve measurement precision and construct depth. Because all variables are self-reported, future studies should incorporate objective or multi-source data to reduce bias. Moreover, because this study relied on a single wave of data, it cannot establish causal relationships. Future research should employ longitudinal designs with appropriate temporal separation or utilize experimental approaches to provide stronger causal evidence. In addition, because respondents were nested within organizations, observations may not have been entirely independent. Although the primary analyses focused on individual-level relationships, future research could employ multilevel modeling or cluster-robust estimation to account more explicitly for potential organizational clustering.

Second, the study focuses on two mediating mechanisms—WIS and WOS. Although these pathways are important, other mechanisms may also link POC to AOC. Future studies should examine additional mediators, such as motivational, cognitive, or identity-based processes, to provide a more comprehensive account of how competitive environments influence commitment. Future research may also explore whether the proposed mechanisms vary across specific job functions, organizational roles, or hierarchical levels, thereby identifying potential boundary conditions of the proposed model.

Third, the unexpected moderation effect of autonomy highlights the need to clarify when autonomy functions as a resource and when it functions as a demand. Although prior research questions its effectiveness under competitive pressure (Jang & Kim, 2025), the present findings suggest that its role depends on both individual and contextual factors. Future studies should incorporate variables such as self-efficacy, competitiveness, and role orientation to better specify these boundary conditions (Fletcher et al., 2008).

Finally, the sample consists primarily of female employees in managerial roles, limiting the generalizability of the findings to non-managerial employees. Although the managerial position was included as a control variable and the results remained largely unchanged, managerial roles involve unique responsibilities and work demands. Future research should examine whether these relationships extend to non-managerial employees, different occupational groups, and a broader range of industries. Primary data collection may also provide greater flexibility in measurement. Additionally, the data were drawn from South Korea, where hierarchical organizational structures and strong performance expectations may shape employees’ experiences of competition and autonomy. Future studies should examine whether the observed relationships replicate in countries characterized by different cultural values and organizational practices.

## Figures and Tables

**Table 1:** Means, standard deviations, and correlations of study variables.

Variable	Mean	SD	1	2	3	4	5	6	7	8	9
1. Age ^1^	44.08	5.50	-								
2. Education level ^2^	2.93	0.85	0.039	-							
3. Marital status ^3^	0.75	0.42	0.103 **	−0.003	-						
4. Position ^4^	2.51	1.08	0.286 **	0.174 **	0.020	-					
5. POC	2.75	0.78	−0.034	−0.002	−0.008	0.012	-				
6. WIS	2.31	0.81	−0.023	0.011	−0.081 **	−0.026	0.248 **	-			
7. WOS	2.90	0.87	−0.013	0.029	−0.016	0.034	0.435 **	0.265 **	-		
8. JA	3.27	0.92	0.069 *	0.001	0.056 *	0.085 **	–0.232 **	−0.297 **	−0.350 **	-	
9. AOC	3.81	0.78	0.264 **	0.056 *	0.138 **	0.160 **	−0.163 **	−0.309 **	−0.159 **	0.265 **	-

Notes. ^1^ Age was measured in years; ^2^ education level was coded as 1 = high school, 2 = college, 3 = bachelors, 4 = masters, 5 = PhD; ^3^ marital status was dummy-coded (0 = single, divorced, or widowed, 1 = married); ^4^ position was coded as 1 = assistant manager, 2 = manager, 3 = deputy manager, 4 = general manager, 5 = executive; N = 1325. * *p* < 0.05, ** *p* < 0.01. SD: standard deviation, POC: performance-oriented competition, WIS: workplace interpersonal strain, WOS: work overload strain, JA: job autonomy, AOC: affective organizational commitment.

**Table 2:** Linear regression analysis results.

Variable	Dependent Variable								
	WIS			WOS			AOC		
	*b*	95% CI		*b*	95% CI		*b*	95% CI	
		LLCI	ULCI		LLCI	ULCI		LLCI	ULCI
Age	0.001	−0.008	0.008	−0.001	−0.009	0.007	0.031 ***	0.024	0.038
Education level	0.016	−0.035	0.066	0.026	−0.024	0.076	0.034	−0.011	0.079
Marital status	−0.150 **	−0.250	−0.050	−0.025	−0.123	0.074	0.164 ***	0.075	0.253
Position	−0.023	−0.065	0.019	0.021	−0.020	0.062	0.061 **	0.024	0.098
POC	0.261 ***	0.206	0.315	0.482 ***	0.428	0.536	−0.064 *	−0.119	−0.010
WIS							−0.249 ***	−0.298	−0.201
WOS							−0.055 *	−0.104	−0.005
R^2^	0.069			0.191			0.187		
F value	19.508 ***			62.209 ***			43.368 ***		

Note. *** *p* < 0.001, ** *p* < 0.01, * *p* < 0.05; *b* = unstandardized coefficient; POC: performance-oriented competition, WIS: workplace interpersonal strain, WOS: work overload strain.

**Table 3:** Results of mediation analysis.

Path	Effect	SE		t-Value	*p*-Value	95% CI		
						LLCI		ULCI
Total effect								
POC → AOC	−0.156	0.026		−6.022	*p* < 0.001	−0.206		−0.105
Direct effect								
POC → AOC	−0.064	0.028		−2.311	*p* < 0.05	−0.119		−0.010
Indirect effect								
Path	Effect		BootSE		BootLLCI		BootULCI	
POC → WIS → AOC	−0.065		0.010		−0.087		−0.046	
POC → WOS → AOC	−0.026		0.012		−0.051		−0.002	
Indirect effect contrast								
WIS-WOS	−0.039		0.017		−0.072		−0.005	

Note. Unstandardized coefficients are reported; Bootstrap samples = 5000; POC: performance-oriented competition, WIS: workplace interpersonal strain, WOS: work overload strain, AOC: affective organizational commitment.

**Table 4:** Results of moderation analysis.

Variable	Effect	SE	t	95% CI	
				LLCI	ULCI
Outcome: Workplace Interpersonal Strain					
POC	0.200	0.028	7.166 ***	0.145	0.254
JA	−0.221	0.024	−9.320 ***	−0.267	−0.174
POC × JA	0.008	0.026	0.307	−0.043	0.059
Outcome: Work Overload Strain					
POC	0.411	0.027	15.181 ***	0.358	0.464
JA	−0.250	0.023	−10.876 ***	−0.296	−0.205
POC × JA	0.056	0.025	2.208 *	0.006	0.105
Levels of JA					
−1 SD	0.360	0.036	9.919 ***	0.289	0.431
Mean	0.411	0.027	15.181 ***	0.358	0.464
+1 SD	0.462	0.035	13.180 ***	0.393	0.531

Notes. *** *p* < 0.001; * *p* < 0.05. POC: performance-oriented competition, JA: job autonomy.

## Data Availability

The datasets presented in this article are not readily available because they are owned and managed by the Korea Women’s Development Institute (KWDI) and were used with permission for this study. Requests to access the datasets should be directed to the Korea Women’s Development Institute (KWDI).

## Abbreviations

The following abbreviations are used in this manuscript:

POC	Performance-oriented competition
AOC	Affective organizational commitment
WIS	Workplace interpersonal strain
WOS	Work overload strain
